# TAPINTO: A Novel Algorithm for Tumor-Associated Antigen Prediction Based on Information about Target Overexpression

**DOI:** 10.34133/csbj.0109

**Published:** 2026-05-15

**Authors:** Cheng-Hsun Chuang, Hsiao-Hsuan Huang, Yi-Syuan Wu, Chia-Hung Chen, Shun-Long Weng, Yu-Chi Chiu, Kuang-Wen Liao

**Affiliations:** ^1^ Institute of Molecular Medicine and Bioengineering, National Yang Ming Chiao Tung University, Hsinchu City 30068, Taiwan, ROC.; ^2^ Industrial Development Graduate Program of College of Engineering Bioscience, National Yang Ming Chiao Tung University, Hsinchu 30068, Taiwan, ROC.; ^3^ Department of Biological Science and Technology, College of Engineering Bioscience, National Yang Ming Chiao Tung University, Hsinchu City 30068, Taiwan, ROC.; ^4^ Department of Medical Research, Hsinchu Mackay Memorial Hospital, Hsinchu City 30071, Taiwan, ROC.; ^5^ Department of Medical Research, Hsinchu Municipal MacKay Children’s Hospital, Hsinchu City 30068, Taiwan, ROC.; ^6^ Department of Medicine, MacKay Medical College, New Taipei City 25245, Taiwan, ROC.; ^7^ MacKay Junior College of Medicine, Nursing and Management College, Taipei City 11260, Taiwan, ROC.; ^8^ Department of Obstetrics and Gynecology, Hsinchu MacKay Memorial Hospital, Hsinchu City 30071, Taiwan, ROC; ^9^ Department of Obstetrics and Gynecology, Hsinchu Municipal MacKay Children’s Hospital, Hsinchu City 30068, Taiwan, ROC.; ^10^ Department of Internal Medicine, Taoyuan General Hospital, Ministry of Health and Welfare, Taoyuan, Taiwan, ROC.; ^11^ Department of Nursing, Yuanpei University of Medical Technology, Hsinchu, Taiwan, ROC.; ^12^ Center for Intelligent Drug Systems and Smart Bio-Devices (IDS^2^B), National Yang Ming Chiao Tung University, Hsinchu City 30068, Taiwan, ROC.; ^13^ Drug Development and Value Creation Research Center, (College of Dental Medicine, Kaohsiung Medical University School of Dentistry), (Graduate Institute of Medicine, College of Medicine), Center for Cancer Research, Kaohsiung Medical University, Kaohsiung City 807378, Taiwan, ROC.; ^14^ Department of Biotechnology and Bioindustry Sciences, College of Bioscience and Biotechnology, National Cheng Kung University, Tainan City 701401, Taiwan, ROC.

## Abstract

Identifying the tumor-associated antigens (TAAs) overexpressed in a subgroup of tumor patients is a substantial challenge for cancer treatment. Although there are several methods based on the concept of differential expression, there is a lack of proper algorithms based on the heterogeneous transcriptome expression for exploring effective TAAs. Here, we propose an algorithm, TAPINTO, to objectively predict overexpressed TAAs whose expression is heterogeneous in cancer patients. This algorithm exploits the dispersion of expression in a subgroup of patients to create 3 quantitative parameters (the specific average expression, frequency, and fold change) for evaluating potential TAAs and has a good performance compared with other approaches. Based on these parameters, TAPINTO successfully identified HER2, a famous therapeutic target, and other potential TAAs (CXCL9, KCNJ3, SQLE, MMP11, and SLC7A2) in breast cancer; moreover, these parameters were dramatically consistent with the trend of clinical outcomes (objective response rate, progression-free survival, and serious adverse effects) of therapeutic antibodies. The ability of TAPINTO to capture heterogeneous expression patterns among patients was further validated in cancer hallmarks, subtypes, and prognosis. This study suggests that this novel method will enable potential TAAs to facilitate the subgroup of patients for diagnosis, prognostication, and therapy to overcome the tumor heterogeneity.

## Graphical Abstract

**Figure:**
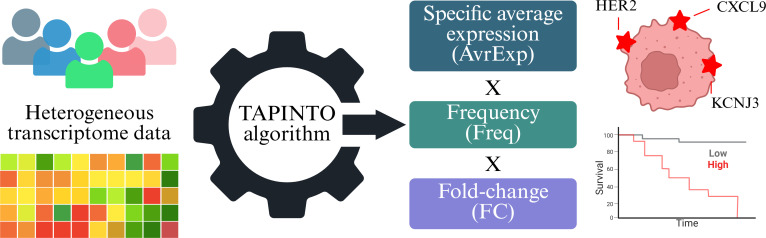

## Introduction

Membrane-associated proteins (MAPs) are overexpressed around the plasma membrane and trigger several cancer hallmarks, such as proliferation, angiogenesis, and metastasis [1–5]. These MAPs can be considered potential tumor-associated antigens (TAAs) and molecular targets in precision medicine [6]. However, the heterogeneous gene expression (HGE) between patients with cancer is a crucial cause of therapeutic failure [7,8]^.^ Therefore, discovering heterogeneous oncogenic characteristics of the patient population is necessary for improving prognostication and therapy [9]. For example, human epidermal growth factor receptor 2 (HER2), a well-known TAA, is overexpressed in 15% to 30% of breast cancer patients, and these patients can benefit from anti-HER2 antibody–drug therapy through antibody-dependent cell-mediated cytotoxicity (ADCC), complement-dependent cytotoxicity (CDC), and antibody-dependent cellular phagocytosis (ADCP) [10–12]. In contrast, approximately 70% to 85% of patients without HER2 overexpression gain no therapeutic benefit from trastuzumab (an anti-HER2 antibody) treatment [13]. Therefore, exploring more potential TAAs as clinical targets for heterogeneous populations is necessary for overcoming the cancer treatment bottleneck [14].

In recent years, bioinformatics strategies have been used to predict potential TAAs via analysis of the differential expression between tumor and adjacent normal samples [15,16]. Based on the average expression level, differentially expressed genes (DEGs) in the whole population are identified by *t* test-based calculations, such as the significance analysis of microarrays (SAM) algorithm and linear models for microarray data (LIMMA) [17,18]. Although calculating average expression is a suitable method for homogeneous conditions, its precision is decreased under the heterogeneous expression conditions within all cancer patients [19,20]. Previous studies have described that TAAs, such as HER2, epidermal growth factor receptor [EGFR; typically overexpressed in basal-like triple-negative breast cancer (TNBC)], programmed death ligand 1 (PD-L1; an immunotherapy target in TNBC), and fibroblast growth factor receptor (FGFR; a driver of resistance in luminal and mesenchymal TNBC subtypes), are often expressed heterogeneously in the patient populations; therefore, these methods might not be suitable for TAA prediction [21–23]. Recently, considering the HGE of subgroup patients, maximum-ordered subset T-statistics (MOST) and detection of imbalanced differential signal (DIDS) were employed to identify outliers in the whole population to assess DEGs [20,24]. However, some studies have reported that MOST and DIDS have a higher false positive rate for DEG detection than other methods [20,25,26].

Most targeted therapies in the clinical trials fail due to insufficient efficacy or safety, suggesting that the exploratory processes to identify ideal targets are still lacking [27]. In addition, tumor evolution in different individuals with unique genetic backgrounds will lead to distinct transcriptomes and result in highly HGE [28]; thus, selection of a universal TAA is difficult. Currently, the published literature has demonstrated that more critical factors should be considered in target selection, including tumor specificity, level of expression, and the number of patients with antigen-positive cancer [14,29,30]. Although several modified methods have improved the power of conventional methods, they still focus on one characteristic (a significant change in the expression level) for DEG detection [19,26]. Nevertheless, biologists do not have a reliable computational method to directly evaluate whether a potential TAA is an ideal target by simultaneously considering multiple dimensions.

In this study, we developed a novel algorithm for TAA prediction based on information about target overexpression (TAPINTO), which enables the objective identification of gene overexpression in a heterogeneous patient population. Because it selects a subgroup of samples dispersed throughout the whole population, TAPINTO can assess 3 important factors for specific subgroup samples: the average expression (AvExp), fold change (FC), and frequency (Freq). Assessment of these factors enables good prediction of specific TAA overexpression. To rationally identify subgroup samples with overexpressed TAAs, HGE is assessed, and the pattern might follow a long-tailed distribution rather than a normal distribution; therefore, TAPINTO can precisely evaluate the abnormal expression of tumor samples. Dramatically, TAPINTO can not only evaluate potential TAAs but also correlate possible clinical outcomes. According to an analysis of breast invasive carcinoma (BRCA) samples from The Cancer Genome Atlas (TCGA) database, TAPINTO in combination with alluvial analysis or other bioinformatics analyses efficiently identified 6 potential TAAs, including HER2, that had distinct expression profiles in subtypes of breast cancer and have been reported to have clear functions in tumorigenesis. In addition, the parameters derived from TAPINTO were also associated with the tumor characteristics and subtypes with the overexpressed genes via enrichment analysis. Overall, TAPINTO, as a novel method, can enable exploitation of potential TAAs and might improve the development of antitumor drugs, clinical therapy, diagnosis, and prognostication in the future.

## Methods

### Acquisition of RNA-sequencing data and data preprocessing

We obtained RNA-sequencing (RNA-seq) data for 19,814 protein-coding genes annotated by GENCODE V22 (https://www.gencodegenes.org/) from 1,109 BRCA samples and 113 adjacent normal samples from the TCGA database (https://portal.gdc.cancer.gov/). These mRNA expression data from TCGA were fragment per kilobase per million (FPKM) normalized. Because most MAPs respond by transmitting biological signals within the plasma membrane to regulate intrinsic or extrinsic cell activity, MAP-coding genes were selected as potential TAAs to constitute the list of candidates for this study [31]. The COMPARTMENTS database, which is a comprehensive resource for protein subcellular localization information, provided a confidence score (3 to 5) for the likelihood that each gene in our list of MAP-coding genes was associated with the plasma membrane [32]. Based on the selection above, the 6,005 protein-coding genes were defined as MAPs. Finally, we generated a matrix of the transcriptomic expression profiles with 6,005 genes in the rows and 1,109 tumor samples and 113 normal samples in the columns.

### Distribution pattern fitting of the empirical data

To fit the data with a long-tailed distribution to our empirical data, we used the R package poweRlaw to fit the gene expression data in this study [33]. The major long-tailed distributions, including exponential, power-law, and log-normal distributions, were used to analyze the gene expression patterns. Before the fitting test, we screened the 6,005 candidate genes based on an average expression level lower than the basic level (<1 FPKM) to avoid noise [34]. Ultimately, 3,661 candidates from 1,109 tumor samples were used to test which gene expression values rejected the null hypothesis. The null hypothesis for the poweRlaw package is “the data are generated from power-law/exponential/log-normal distribution”, and 0.1 was used as the *P* value threshold in this study [35].

### Algorithm for identifying samples with overexpression and parameter calculation

Unlike in a homogeneous population, in a heterogeneous population, samples with overexpression should be distinguished and separated from normal-like samples. To separate the 2 subgroups for each gene candidate, we calculated the standard deviation (SD) to detect the change in expression dispersion by following 3 steps [Eqs. (1) to (5)]. First, the expression of each gene was rearranged in descending order [Eq. (1)]. Second, the SDs were calculated with continuous sampling from the first to third sample, to the fourth sample, to the fifth sample, and so on [Eqs. (2) to (4)]. Third, the maximum SD value was set as the cutoff point to separate the 2 heterogeneous subgroups [Eq. (5)]. Finally, the samples whose values were higher than or equal to the sample at the maximum value were considered the samples with overexpression.

### Index scoring method and alluvial diagram analysis

In the identified subgroup of tumor samples, we calculated the FC, AvExp, and Freq values [Eqs. (6) to (8)]. We performed alluvial diagram analysis to examine the effect of the 3 parameters on TAA identification and present the *P* value with the R package ggalluvial. The *P* values were calculated by one-tailed Wilcoxon rank-sum tests and adjusted for multiple testing with the Benjamini–Hochberg method to control the false discovery rate (FDR). The continuous values of the FC, AvExp, and Freq for each gene candidate were transformed into a percentile scale and separately set as class intervals in the alluvial strata. For the adjusted *P* value, the significance threshold was set at 0.05, and results are presented as not significant (n.s.) or *P* < 0.05. For clinical outcome analysis, we constructed a list of well-known therapeutic targets according to the ClinicalTrials.gov website (https://clinicaltrials.gov/). The keywords used to search the clinical studies included the following: [available, active, not recruiting, completed, suspended, terminated, or withdrawn Studies]; [studies with results]; [interventional studies]; [breast cancer]; and [phase 3, 4]. For alluvial analysis, each gene with a nonsignificant *P* value was given a zero value, and each gene with a significant *P* value was given one point. For the 3 critical factors, each gene ranking in the top 25% was given 2 points, each gene ranking in the top 25% to 50% was given one point, and each gene ranking in the top 50% to 100% was given a zero value. The parameters were equally weighted to provide a balanced integration of prevalence, expression intensity, and specificity, with quartile-based thresholds applied to categorize the TAA potential of candidates. Then, the index score was calculated as the index point of the *P* value multiplied by the sum of the index points of the other 3 critical factors. Ultimately, the highest index score was 6 points, and the lowest index score was zero.

### Functional and disease enrichment analyses

Gene set enrichment analysis (GSEA) using the clusterProfiler R package [36] was performed with the 6,005 candidate genes. For GSEA, gene sets were downloaded from the MSigDB database of the Broad Institute [37], and hallmark gene sets were used for quantification of pathway enrichment. disease ontology semantic and enrichment analysis (DOSE) was performed with the DOSE R package [38] on the candidates with TAA scores in the top 10% [Eq. (9)]. The *P* value of both enrichment analyses was calculated based on 10,000 permutations and adjusted for multiple testing with the Benjamini–Hochberg procedure to control the FDR. The cutoff *P* values for the GSEA and DOSE were at 0.005 and 0.05, respectively. The R package enrichplot [39] employs many visualization methods to help interpret enrichment results and was applied to visualize the GSEA and DOSE results for the TAAs in breast cancer.

### Logistic regression and overall survival analyses

The logistic regression and survival analyses were processed by the stats and survival packages of the R programming language, respectively. A total of 1,097 BRCA samples annotated with survival status (alive or dead) and follow-up (months) data from TCGA were used for the overall survival (OS) analysis. To predict the risk of survival, we used the survival status associated with the BRCA samples as the response value and the expression of potential TAAs as the variables to build the logistic regression model. Based on the median risk scores calculated by the logistic regression analysis, the samples were separated into a high-risk and low-risk group. For the survival analysis, the log-rank test was used to test the significance of differences in follow-up between the 2 groups, and a *P* value less than 0.05 was considered to indicate statistical significance.

### Evaluation of TAPINTO performance

To evaluate the accuracy of TAPINTO for identifying samples with overexpression, we selected 731 samples with HER2 immunohistochemistry (IHC) status (“HER2 Positive” or “HER2 Negative”) data from 1,109 BRCA samples and ordered them from high to low mRNA expression. Then, we calculated the positive predictive value (PPV = No. of true positive/No. of predicted positives) and negative predictive value (NPV = No. of true negative/No. of predicted negatives) for each. To compare the performance of TAPINTO with that of well-known TAA prediction methods (*t* test, LIMMA, and DIDS), the FC was calculated and used for comparisons due to its wide usage in most methods. In addition, we constructed a list of well-known breast cancer TAAs via a literature review (Table S1). These TAAs included receptor tyrosine kinases (RTKs), carcinoembryonic antigens (CEAs), mucins (MUCs), Frizzled receptors (FZDs), and matrix metalloproteinases (MMPs). The FC of the *t* test method was calculated as the average expression of tumor samples/the average expression of adjacent normal samples. For LIMMA and DIDS, the FC was obtained by using R packages and/or source codes (LIMMA: https://bioconductor.org/packages/release/bioc/html/limma.html; DIDS: https://github.com/NKI-CCB/dids). In the DOSE analysis, the FC values of LIMMA and DIDS were compared with those of the TAA score. To evaluate sensitivity and specificity simultaneously, accuracy analysis was implemented via the pROC R package [40], which was used to plot receiver operating characteristic (ROC) curves and calculate the area under the ROC curve (AUC) values.

Algorithm equations

*S* is the number of sample(s) ranked according to highest to lowest expression.

s=1,2,3,4,5,…,N (1)

$x_{i}$ is the expression for the sample i = 1, 2, 3 …,s.

${\bar{x}}_{s}\;$ is the average expression of s samples.

$${\bar{x}}_{s}=\frac{\sum_{i=1}^{s}x_{i}}{s}$$ (2)

$\bar{y}\;$is the average expression of normal samples.

$$\bar{y}=\frac{\sum_{i=1}^{N}y_{i}}{N}$$ (3)

$S\left(s\right)$ is the SD of s samples.

$$\mathrm{SD}\left(s\right)=\sqrt{\frac{\sum_{i=1}^{s}{\left(x_{i}-{\bar{x}}_{s}\right)}^{2}}{s-1}}$$ (4)

$\hat{s}$ is the maximum SD of s.

$$\hat{s}=\text{arg}\;\underset{0\leq s\leq N}{\text{max}}\mathrm{SD}\left(s\right)=\sqrt{\frac{\sum_{i=1}^{s}{\left(x_{i}-{\bar{x}}_{s}\right)}^{2}}{s-1}}$$ (5)

Freq is the frequency of high expression subgroup (outlier)in the total tumor samples.

$$\text{Freq}=\frac{\hat{s}}{N}$$ (6)

AvrExp is the average of the high expression subgroup (outlier) in the total tumor samples.

$$\text{AvrExp}={\bar{x}}_{\hat{s}}=\frac{\sum_{i=1}^{\hat{s}}x_{i}}{\hat{s}}$$ (7)

FC is the fold change between the high expression tumor subgroup (outlier)and the normal samples.

$$\mathrm{FC}=\frac{\text{AvrExp}}{\bar{y}}$$ (8)

TAAscore is the integration form of the 3 critical factors.

TAAscore=AvrExp×FC×Freq (9)

## Results

### The expression of TAA mRNA was heterogeneous within the breast tumors of patients

**Fig. 1.**
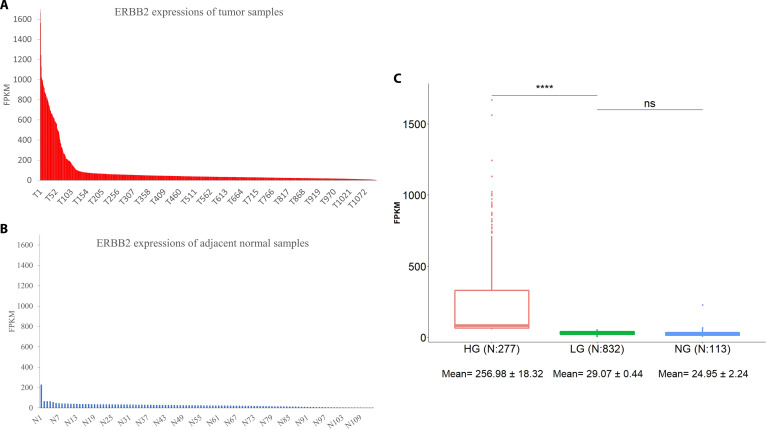
The mRNA expression levels of HER2 in breast cancer patients. (A) The HER2 expression levels in 1,109 breast cancer tumor samples were ranked, and the samples are presented from high to low expression. (B) The HER2 expression levels in 113 tumor-adjacent normal samples were also ranked, and the samples are presented from high to low expression. (C) Comparison of HER2 expression between 3 distinct groups [HG: high-expression tumor group, top 25% of breast cancer samples (*N* = 277); LG: low-expression tumor group, remaining 75% of breast cancer samples (*N* = 832); NG: adjacent normal group (*N* = 113)]. ****P < 0.001; ns (not significant): *P* > 0.05.

Heterogeneity of mRNA expression exists in cancer patients. The mRNA expression of ERBB2 (also as known as HER2) in breast cancer tissues and adjacent normal tissues was arranged in descending order (Fig. 1A and B). The results showed that a subgroup with high expression existed in the tumor population but not within the adjacent normal group. Because HER2 is commonly overexpressed in a quarter of breast cancer patients, we set the breast cancer samples with expression in the top 25% as the high-expression subgroup, and the remaining 75% of tumor samples were grouped in the low-expression subgroup. The average expression in the adjacent normal group was 24.95 ± 2.24 FPKM, which was similar to the average of the low-expression subgroup (approximately 29.07 ± 0.44 FPKM). However, both were significantly lower than that in the high-expression subgroup (Fig. 1C). This result indicated that heterogeneity of TAA expression existed in the tumor samples, and most tumor samples were not significantly different from the adjacent normal group. However, the normal-like tumor subgroup with lower gene expression may interfere with TAA prediction accuracy in methods based on the whole population.

### The expression of most plasma membrane protein-coding genes follows a long-tailed distribution

**Fig. 2.**
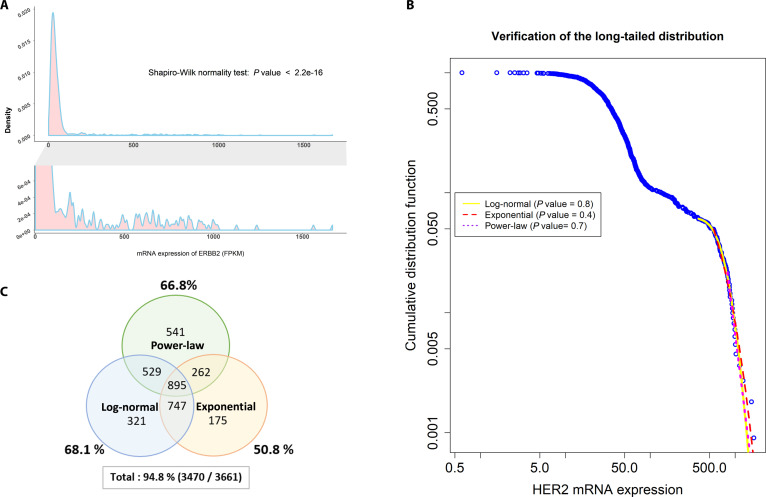
Gene expression distribution in breast cancer samples. (A) The probability of the HER2 expression pattern having a normal distribution was determined by the Shapiro–Wilk normality test. The results revealed that HER2 expression in the breast cancer samples was not normally distributed. (B) The expression of HER2 in the 1,109 breast cancer samples is presented by the strength of HER2 expression (FPKM, *x* axis) and the cumulative distribution function (*y* axis), and the values are presented as open blue circles. A long-tailed distribution of HER2 expression was verified with tests, and a log-normal distribution (yellow color), an exponential distribution (red color), and a power-law distribution (purple color) and their expected values are labeled by different color lines. The *P* values were calculated by the poweRlaw package of R. (C) The Venn diagram shows the numbers of genes with expression fitting a long-tailed distribution (of 3,661 genes; average expression ≥ 1 FPKM; 6,006 plasma membrane protein-coding genes for breast cancer were considered); a log-normal distribution, an exponential distribution, or a power-law distribution. The number of genes and the corresponding percentage are labeled in each group in the Venn diagram.

Furthermore, the expression of ERBB2 was transformed into a probability density function (PDF) to visualize the distribution (Fig. 2A), and the distribution was clearly not following a normal distribution after testing with the Shapiro–Wilk method (*P* < 0.05). Unlike the normal distribution, this distribution seems to be positively skewed (Fig. 2A, bottom). We proposed a hypothesis that the expression of ERBB2 follows a long-tailed distribution with a gradually decreasing tail. To verify this hypothesis, the poweRlaw package was used to test whether ERBB2 expression had a long-tailed distribution via the power-law, exponential, and log-normal distribution test methods. As shown in Fig. 2B, all the null hypotheses could not be rejected by the ERBB2 mRNA expression distribution, and therefore, the expression had a long-tailed distribution.

Subsequently, the expression distributions of the remaining plasma membrane protein-coding genes were all tested in the same way. Excluding the genes with no expression (<1 FPKM), 3,661 genes were selected for further statistical analysis. The results revealed that over 50% of the genes could not reject the null hypothesis in each testing method, and the expression of 94.8% of the plasma membrane protein-coding genes had a long-tailed distribution (Fig. 2C). This finding led to the development of a novel method that is crucial for exploring TAAs from plasma membrane-coding genes characterized by expression with a long-tail distribution.

### The high-expression subgroup was defined objectively from a heterogeneous tumor population by calculation of SDs

**Fig. 3.**
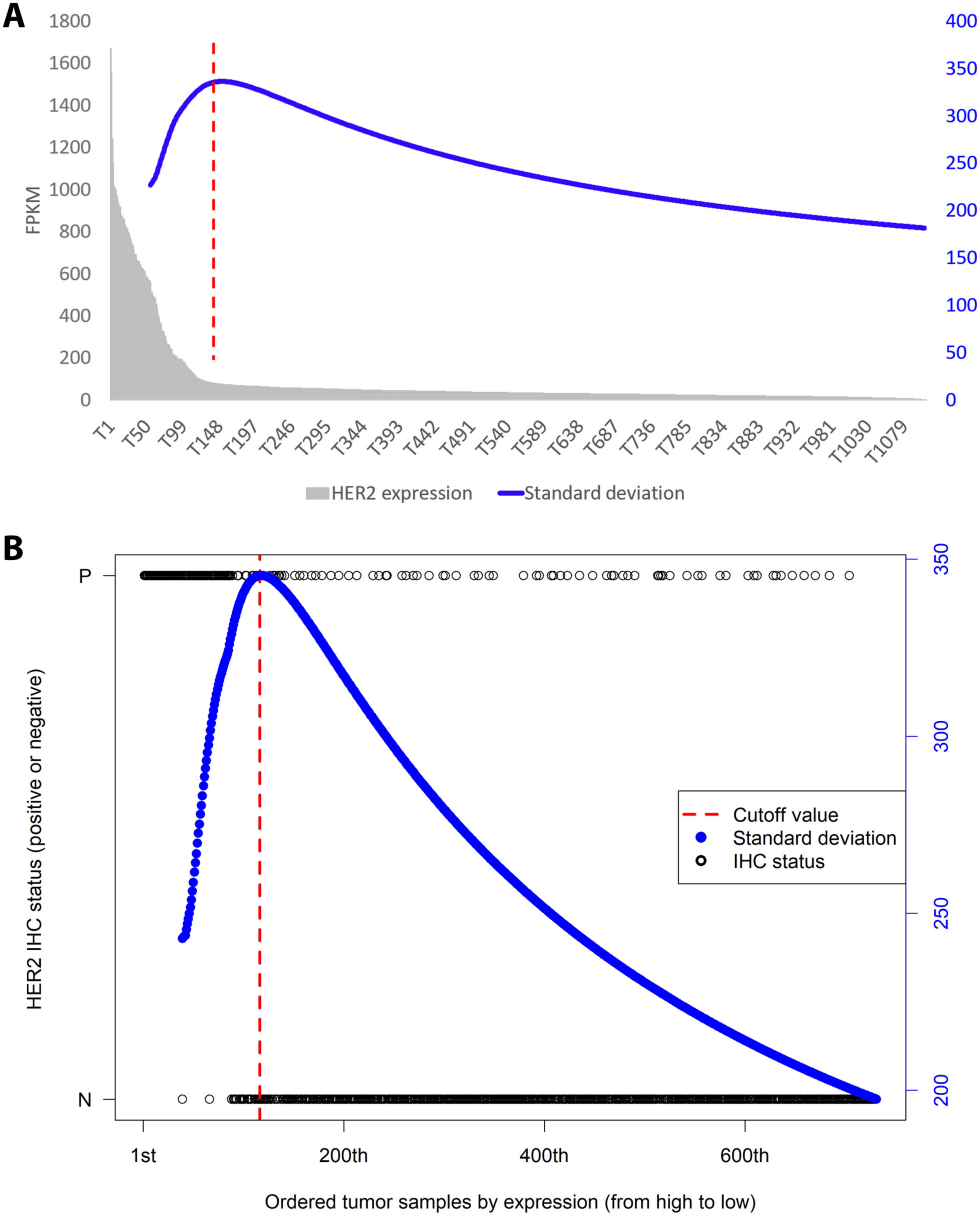
TAPINTO identified a subgroup with high expression of HER2 based on continuous SD values. (A) Original HER2 expression levels (FPKM) of the 1,109 samples ranked from high to low expression (gray bars). Based on these data, the continuous SD values (blue line) were calculated by iteratively including samples from the highest to the lowest expression. The SD increases as high-expression outliers are included and reaches a maximum at the point where additional lower-expression samples begin to reduce the overall dispersion of the cumulative subset. This maximum value (red dashed line) represents the turning point that objectively defines the boundary between the high-expression subgroup and the normal-like tumor subgroup. (B) The HER2 clinical IHC status (black circles) of 731 of the 1,109 breast cancer samples was either positive (P) or negative (N), as shown, and the results are ordered as above. The standard deviation values of 731 breast cancer samples were also calculated and are shown as the blue line, and the maximum value of the standard deviations is indicated by the red dashed line.

Because the high-expression subgroup was far from the central levels of expression of the whole heterogeneous tumor population and showed a distribution similar to a long-tailed distribution, we proposed that a large dispersion of expression existed between the 2 distinct tumor subgroups (the high-expression and normal-like subgroups). To identify the high-expression subgroups for each gene candidate, we designed a formula based on SDs that could present the dispersion of ERBB2 gene expression using 3 steps (see Methods). The ERBB2 mRNA expression of the breast cancer samples was sorted in descending order with [Eq. (1)] (Fig. 3A, black line), and the SD was calculated with [Eq. (4)] (Fig. 3A, blue line). Consequently, the population with the highest SD values was identified with [Eq. (5)] (Fig. 3A, dashed line), and the samples in this identified population accounted for 14% of the total samples (154/1,109) and were classified as BRCA samples with high expression. To determine the consistency of the identified outlier samples with the clinical data and to verify TAPINTO’s performance, the IHC statuses of 731 BRCA samples from the TCGA database were used. The results showed that 16% of the total samples (116/731) had high expression, and the AUC value of this method was 0.87 (the *y* axis shows a positive and negative status with P and N, respectively). In addition, the PPV was 80.2% (predicted positives: 93; true positives: 116), and the NPV was 88.6% (predicted negatives: 545; true negatives: 615) based on IHC detection (Fig. 3B). Notably, TAPINTO identified that 16% of the 731 breast cancer samples overexpressed HER2 (Fig. 3A), consistent with previous clinical studies that indicated that 15% to 25% of the breast cancer population has overexpression of HER2. The TAPINTO method can further calculate and obtain the following parameters: the *P* value, average specific expression, fold change of specific expression, and frequency of specific expression [Eqs. (6) to (8)].

### Validation of the ability of TAPINTO to predict TAAs and comparison of TAPINTO with other methods

**Fig. 4.**
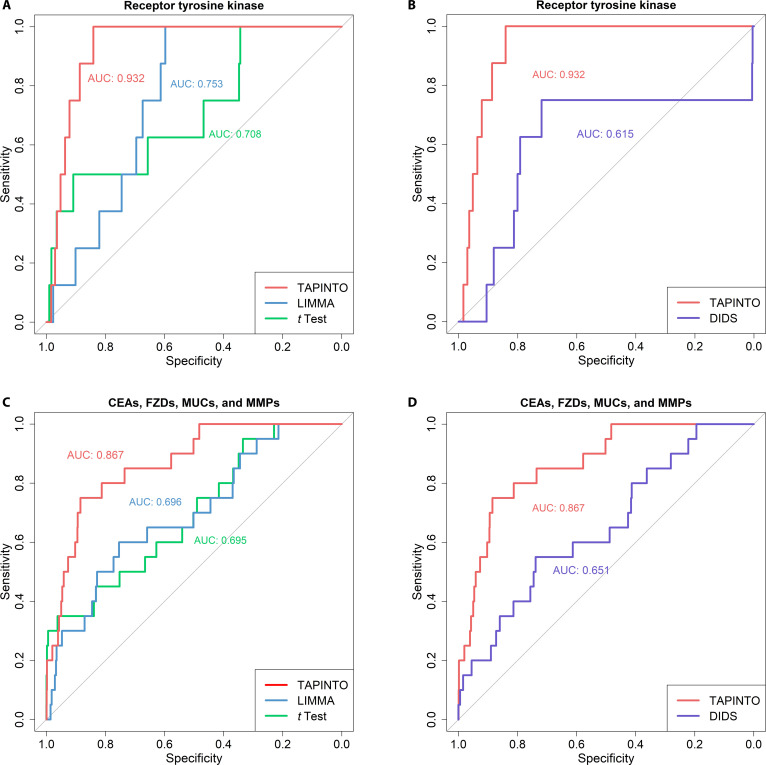
Comparison of the TAA prediction accuracy of TAPINTO and other methods. This figure evaluates the sensitivity of various methods in identifying established TAAs using ROC/AUC analysis. (A) Sensitivity for receptor tyrosine kinase (RTK) TAAs: Comparison of prediction accuracy using TAPINTO-calculated fold change (red line) versus traditional differential expression methods, including LIMMA (blue line) and *t* test (green line), based on well-known breast cancer targets. (B) Comparison with existing outlier detection: Performance benchmarking of TAPINTO against DIDS (deep blue line), a conventional outlier-based detection method, specifically evaluating the ability to identify oncogenic RTK targets within heterogeneous populations. (C and D) Generalizability across diverse antigen families: Evaluation of prediction sensitivity for various TAA categories, including CEA-, MMP-, FZD-, and MUC-encoding genes. TAPINTO’s performance is compared against (C) mean-based statistical tests (LIMMA and *t* test) and (D) the specific fold change logic of DIDS.

In general, TAAs have higher expression in tumor cells than in normal cells, and such differential expression can be quantified by the FC value. Given the heterogeneous expression at the interpatient level, certain TAAs are overexpressed in some subgroups of patients but not the whole population. Figure 1C shows that over 85% of samples did not significantly overexpress HER2 compared to normal samples, suggesting that the whole population’s average expression may not be a proper representation of the expression in a specific subgroup of patients with high TAA expression. By using the TAPINTO method, we can obtain the FC values derived from comparing the high-expression subgroup samples and adjacent normal tissue samples. For the comparison, different methods, such as whole-group-based methods (*t* test and LIMMA) and a subgroup-based method (DIDS), were used, and critical MAP families, such as RTKs, CEAs, MUCs, FZDs, and MMPs (shown in Table S1), were selected to evaluate the accuracy of these distinct methods. The ROC curves showed that TAPINTO had a higher AUC value than the other methods for predicting RTK antigens (TAPINTO: 0.932 versus LIMMA: 0.753 or *t* test: 0.708) and CEA, MUC, FZD, and MMP family antigens (TAPINTO: 0.865 versus LIMMA: 0.695 or *t* test: 0.693) (Fig. 4A and B). In the comparison of the subgroup-based methods, the ROC curves again showed that TAPINTO had a higher AUC value than the DIDS method for predicting both RTK (0.932 versus 0.615) and other family antigens (0.865 versus 0.651) (Fig. 4C and D). Therefore, the TAPINTO method can predict TAAs better than other methods can by using only the FC value.

### Analysis of the TAPINTO parameters as indicators for evaluating potential TAAs and the outcomes of clinical trials

**Fig. 5.**
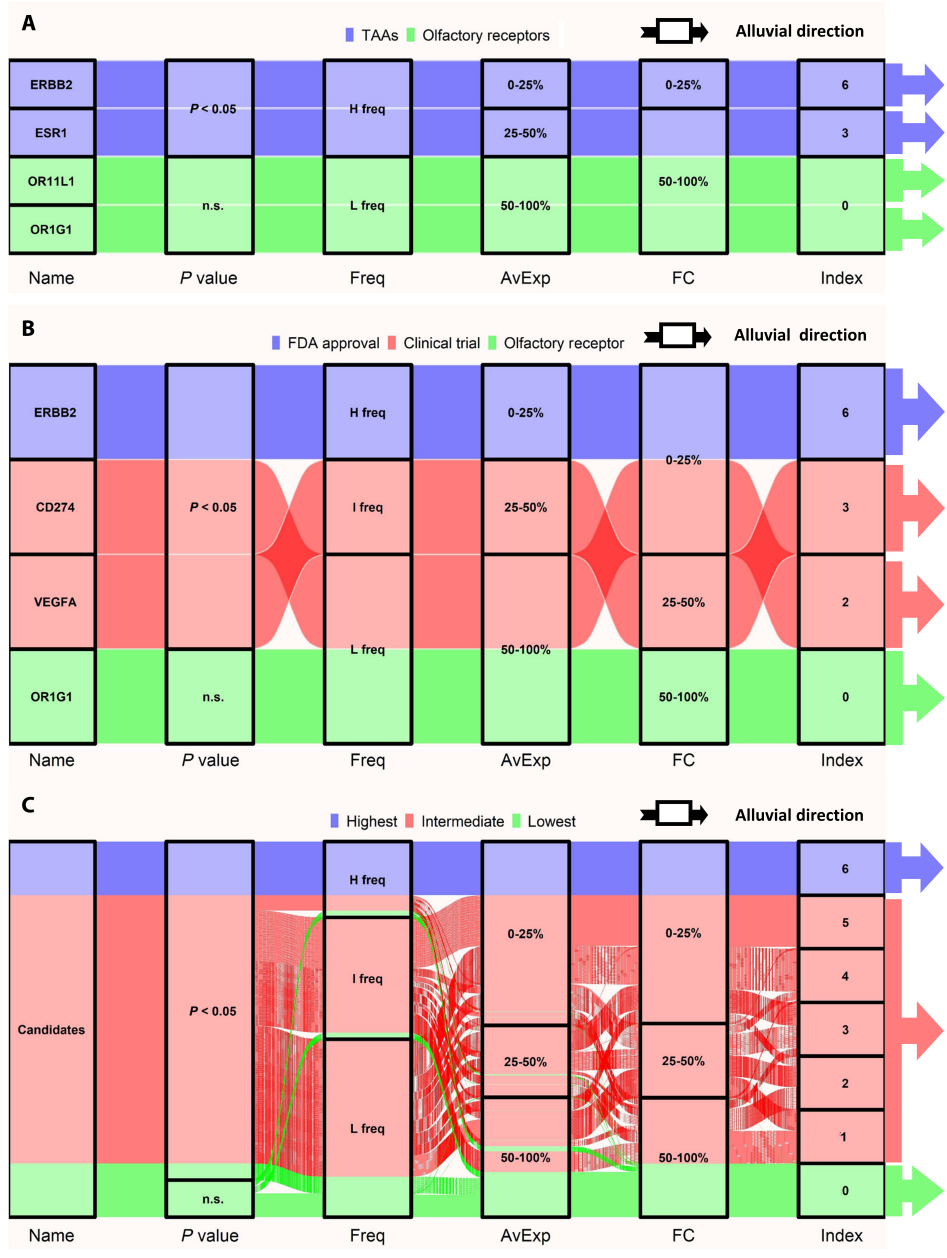
Alluvial diagram of the predicted TAAs for breast cancer based on TAPINTO parameters. The diagram visualizes the integration of various TAPINTO parameters and the resulting classification of gene candidates. The flow from left to right represents the algorithmic progression from individual parameter evaluation to final index scoring.(A) For the evaluation of TAAs, ERBB2 (also known as HER2), ESR1 (also known as ER), and 2 OR-encoding genes (OR11L1 and OR1G1) were considered positive control TAA antigens (HER2 and ER) and normal antigens. The *P* value, frequency (Freq), average expression (AvExp), and fold change (FC), which were calculated with TAPINTO, were used as criteria for TAA evaluation for the characterization of TAAs in the alluvial assay. The quantitative values were added as indexes to evaluate the TAA potential of the test object. (B) The TAPINTO parameters of other therapeutic TAA genes (CD274 and VEGFA) from clinical studies were also evaluated; ERBB2 and OR1G1 were again used as positive and negative controls, respectively. (C) Overall, 5,998 plasma-associated genes excluding the previous 6 genes were tested for their TAA potential. The 5,998 genes were evaluated from different perspectives based on the scale of the parameters and were classified from a score of 6 to 0. Among these, 5 genes (CXCL9, KCNJ3, SQLE, MMP11, and SLC7A2) achieved the highest score of 6 points, the same as ERBB2, and were identified as ideal TAA candidates. The flow direction of the plot is from left to right.

Based on the TAPINTO algorithm, the parameters (the *P* value, average, fold change, and frequency of specific expression) for every gene candidate were obtained with [Eqs. (6) and (7)]. These parameters of each gene can be used to better evaluate and predict TAAs (Supplementary Materials). For testing, drug targets as positive candidates (ERBB2 and ESR1) and olfactory receptor (OR) genes as negative candidates (OR11L1 and OR1G1), which are specifically expressed in cilia of olfactory sensory neurons but not breast cancer cells, were applied in the TAA prediction. Alluvial analysis was used to evaluate the TAA potential of candidate genes with the TAPINTO parameters. Figure 5A shows that ERBB2 had the highest score (6 points), and ESR1 had a moderate score (3 points); both are regarded TAAs targeted by U.S. Food and Drug Administration-approved antitumor drugs in breast cancer.

In contrast, the scores for the 2 ORs (OR1G1 and OR11L1) were the lowest (zero), and thus, they would not be suggested to be TAAs. Therefore, the TAPINTO parameters combined with alluvial analysis are convenient tools for evaluating TAA potential. Subsequently, ERBB2 and OR1G1 were used as the positive and negative controls, respectively, and we further evaluated the TAA potential of CD274 (PD-L1) and vascular endothelial growth factor A (VEGF), which have been considered critical TAAs in many cancers, in breast cancer. Figure 5B shows that VEGFA and CD274 were determined to have scores of 3 and 2, respectively, which suggests that the 2 gene products may not be ideal TAAs, like HER2/ERBB2, but they are still better than OR1G1.

Because the expression of TAAs may be associated with the effects of targeted therapy in the clinic, the results of clinical trials, such as the objective response rate (ORR), progression-free survival (PFS), and serious adverse effects (SAEs), in breast cancer were analyzed (detailed information is shown in Table S2). Specifically, Table 1 shows that Freq positively correlated with ORR (ERBB2: 13.87%, 81.3%; VEGFA: 4.33%, 32.2%; CD274: 0.54%, 9.6%), AvExp positively correlated with PFS (ERBB2: 412.00 FPKM, 9.90 months; VEGFA: 39.38 FPKM, 9.2 months; CD274: 14.37 FPKM, 2.1 months), and FC negatively correlated with SAEs (ERBB2: 16.51, 15.63%; CD274: 12.01, 20.00%; VEGFA: 7.41, 37.57%), suggesting that TAPINTO parameters reflect the likelihood of clinical response, duration of therapeutic benefit, and target safety profile, respectively. These results suggest that assessment of the TAPINTO parameters in combination with alluvial analysis can be used to evaluate the TAA potential of gene candidates and determine clinical outcomes before executing the trial. Furthermore, the TAPINTO platform was used to assess all 5,999 genes (6,005 plasma membrane protein-coding genes excluding 6 previous genes) to determine which genes had potential as ideal TAAs for breast cancer. Figure 5C shows that 5 candidates [C-X-C motif chemokine ligand 9 (CXCL9), potassium inwardly rectifying channel subfamily j member 3 (KCNJ3), squalene epoxidase (SQLE), matrix metallopeptidase 11 (MMP11), and solute carrier family 7 member 2 (SLC7A2)] with the highest index (6 points, the same as ERBB2) were considered ideal TAAs, and the parameters are listed in Table 2. According to the published literature, all the candidate genes are involved in the process of tumorigenesis in different subtypes of breast cancer (Table 3). These results demonstrate that the TAPINTO platform has the ability to identify TAAs from heterogeneous breast cancer samples.

**Table 1.** Information on 3 antibody drugs in clinical trials and their targets and TAPINTO parameters

Gene	Freq (%)		ORR (%)	AvExp (FPKM)		PFS	FC		SAEs	Score
	Scale	Value		Top %	Value		Top %	Value		
ERBB2	H. freq.	13.87	81.30	0–25%	412.00	9.90	0–25%	16.51	15.63%	6
VEGFA	I. freq.	4.33	32.20	25–50%	39.38	9.20	25–50%	7.41	37.57%	3
CD274	L. freq.	0.54	9.60	50–100%	14.37	2.10	0–25%	12.01	20.00%	2

ORR, objective response rate; PFS, progression-free survival; SAEs, serious adverse effects; Freq, frequency; AvExp, average expression; FC, fold change

**Table 2.** The 5 TAPINTO-predicted candidates with the highest score (6 points) from alluvial analysis

	*P* value	Freq	AvExp	FC
KCNJ3	1.7E−44	11.54	83.80	78.40
MMP11	6.4E−67	72.68	82.96	68.05
CXCL9	7.6E−40	10.73	225.48	46.39
SQLE	5.3E−40	10.82	81.91	14.57
SLC7A2	4.8E−04	11.45	155.12	10.79

**Table 3.** The functions of the 5 TAPINTO-predicted TAAs and their relationships with tumors

	Function	Cancer types	Tumorgenicity	Ref.
KCNJ3	Potassium channel	ER^+^ breast cancer	Metastasis	[44]
MMP11	Matrix metalloproteinase	Invasive breast cancer	Metastasis	[49–51]
CXCL9	Cytokine	Hormone receptor^+^ breast cancer	Metastasis	[43,63]
SQLE	Squalene epoxidase	Multiple subtypes of breast cancer	Metastasis	[46–48,64]
SLC7A2	Cationic amino acid transporter	Melanoma	Drug resistance	[54]

### Multiple perspectives assessed the susceptibility of TAPINTO to the characterizations of tumor heterogeneity

**Fig. 6.**
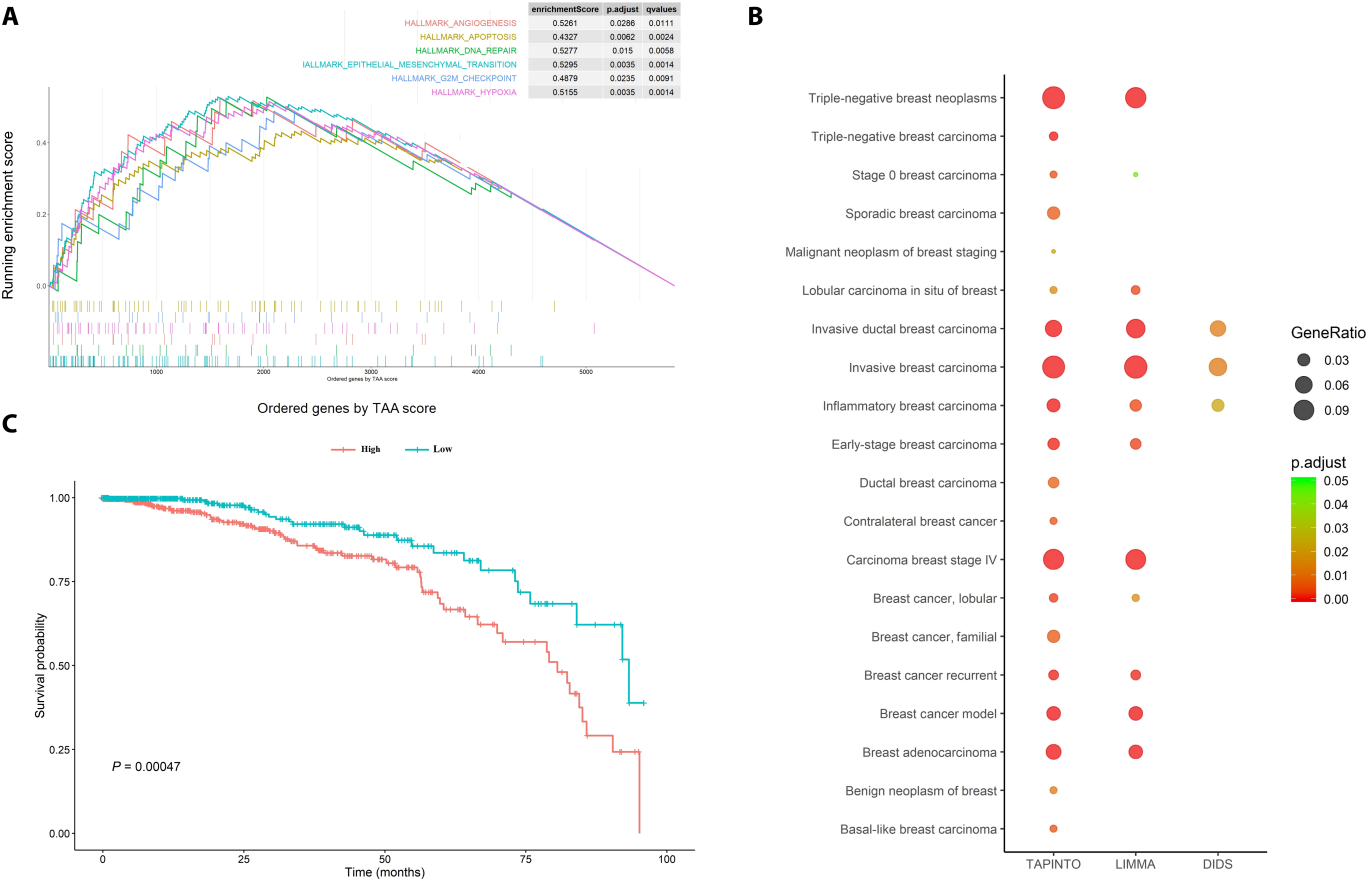
Application of TAPINTO to assess the characterizations of tumor heterogeneity. (A) TAA scores derived from TAPINTO were able to identify oncogenic pathways. The TAA score was used with the gene set enrichment analysis to identify 28 significantly enriched hallmark gene sets associated with tumor progression from MSigDB. The enrichment plots of 6 well-known cancer hallmarks are shown. (B) The disease enrichment analyses of the genes with fold changes in the top 10% calculated by 3 methods (TAPINTO, LIMMA, and DIDS) provided different degrees of identification for subtypes of breast cancer. The circle dots represent the gene ratio, and the *P* values are displayed with colors. (C) The survival risk was analyzed based on TAPINTO and potential TAAs (ERBB2, CXCL9, KCNJ3, MMP11, SQLE, and SLC7A2) that had a score of 6 in the binary logistic regression. The 1,097 breast cancer samples with overall survival (OS) annotation from TCGA were divided into 2 groups: high group (risk scores ≥ median) and low group (risk scores < median). Their OS at 8 years was calculated and displayed in a Kaplan–Meier plot, and the significance was determined by the log-rank test (*P* = 0.00047).

As with previous results, TAPINTO parameters (average specific expression, frequency of specific expression, and fold change of specific expression) were consistent with the trend of clinical outcome from clinical trials; thus, they were further integrated into the TAA score [Eq. (9)]. Subsequently, the TAA scores of genes were ordered and subjected to GSEA. According to the MSigDB database, the set of gene candidates with high TAA scores from breast cancer showed significant enrichment of 28 hallmark gene sets, including 6 well-known cancer-related hallmarks [angiogenesis, DNA repair, epithelial–mesenchymal transition (EMT), apoptosis, hypoxia, and G2M checkpoint] (Fig. 6A and Table 4). Notably, the other 22 pathways could be categorized into 6 classes, all of which correlated with the features of tumorigenesis (Table 4).

**Table 4.** Twenty-eight enriched hallmarks from MSigDB based on the TAA score

Gene sets	setSize	EScore	NES	p.adjust	qvalues
Cancer hallmark					
HALLMARK_EPITHELIAL_MESENCHYMAL_TRANSITION	103	0.5295	1.858	0.0041	0.0016
HALLMARK_DNA_REPAIR	24	0.5277	1.5782	0.0164	0.0063
HALLMARK_ANGIOGENESIS	20	0.526	1.5274	0.0199	0.0077
HALLMARK_HYPOXIA	64	0.5154	1.741	0.0041	0.0016
HALLMARK_G2M_CHECKPOINT	28	0.4878	1.4996	0.029	0.0112
HALLMARK_APOPTOSIS	72	0.4327	1.4798	0.0057	0.0022
Oncogene-related gene sets					
HALLMARK_MYC_TARGETS_V1	20	0.5697	1.6542	0.0074	0.0028
HALLMARK_MTORC1_SIGNALING	63	0.5487	1.8521	0.0041	0.0016
HALLMARK_P53_PATHWAY	80	0.4749	1.6378	0.0041	0.0016
HALLMARK_TNFA_SIGNALING_VIA_NFKB	77	0.4206	1.4456	0.0093	0.0036
HALLMARK_IL2_STAT5_SIGNALING	117	0.3690	1.3093	0.0149	0.0058
HALLMARK_KRAS_SIGNALING_UP	93	0.3662	1.2778	0.0385	0.0149
Metabolism-related gene sets					
HALLMARK_GLYCOLYSIS	56	0.5345	1.7832	0.0041	0.0016
HALLMARK_FATTY_ACID_METABOLISM	43	0.5288	1.7184	0.0041	0.0016
HALLMARK_CHOLESTEROL_HOMEOSTASIS	37	0.5179	1.6517	0.0058	0.0022
HALLMARK_XENOBIOTIC_METABOLISM	70	0.4363	1.4881	0.0058	0.0022
Immune-related gene sets					
HALLMARK_INTERFERON_ALPHA_RESPONSE	29	0.5278	1.6292	0.0058	0.0022
HALLMARK_TNFA_SIGNALING_VIA_NFKB	77	0.4206	1.4456	0.0093	0.0036
HALLMARK_INTERFERON_GAMMA_RESPONSE	78	0.4098	1.4103	0.0111	0.0043
HALLMARK_ALLOGRAFT_REJECTION	117	0.3988	1.4151	0.0041	0.0016
HALLMARK_COMPLEMENT	109	0.3663	1.2927	0.0207	0.0080
Hormone-related gene sets					
HALLMARK_ESTROGEN_RESPONSE_LATE	83	0.5719	1.9798	0.0041	0.0016
HALLMARK_ESTROGEN_RESPONSE_EARLY	90	0.5408	1.8812	0.0041	0.0016
HALLMARK_ANDROGEN_RESPONSE	44	0.4771	1.5572	0.0075	0.0029
Damage response-related gene sets					
HALLMARK_UNFOLDED_PROTEIN_RESPONSE	21	0.6475	1.8899	0.0041	0.0016
HALLMARK_UV_RESPONSE_UP	60	0.4470	1.4992	0.0058	0.0022
Environment-related gene sets					
HALLMARK_APICAL_SURFACE	39	0.5026	1.6139	0.0075	0.0029
HALLMARK_COAGULATION	75	0.4896	1.6791	0.0041	0.0016
HALLMARK_PROTEIN_SECRETION	44	0.5000	1.6320	0.0041	0.0016

Moreover, the TAA score could also be used to assess the tissue features and molecular subtype of heterogeneous tumors through DOSE. Figure 6B shows that those gene clusters with a high TAA score and high gene ratio were significantly enriched in aggressive breast cancer samples, including triple-negative breast neoplasm, invasive ductal breast carcinoma, invasive breast carcinoma, and stage IV breast carcinoma samples. Consistently, these results were derived from TCGA, which includes invasive breast carcinoma data. Moreover, the tissue subtype, malignant features, and molecular subtype of these heterogeneous invasive breast cancer samples could be further analyzed, including analysis of pathologic type (sporadic breast carcinoma, ductal breast carcinoma, basal-like breast carcinoma, and contralateral breast cancer), stage (benign breast neoplasm, stage 0 breast carcinoma, early-stage breast cancer, and breast malignant neoplasm), and molecular type (triple-negative breast carcinoma and familial breast cancer). These results suggest that TAPINTO parameters has a susceptibility for the heterogeneity of potential oncogenic pathway gene sets, of breast cancer subtypes, and of prognosis. The other methods LIMMA and DIDS could also identify some cancer types (invasive breast carcinoma, invasive ductal breast carcinoma, and inflammatory breast carcinoma) but not as well as TAPINTO could (Fig. 6B).

To employ the TAPINTO parameters for survival analysis (in terms of OS), a signature of the 6 candidate genes was used to predict the survival risk of breast cancer patients, and logistic regression was used to calculate the risk scores of the breast cancer samples. As seen in Fig. 6C, the samples with high risk scores had a significantly poorer survival prognosis at 8 years (*P* = 0.00047; Fig. 6C). The results indicate that the 6 potential TAAs, which are predicted by TAPINTO, might play an important role in the progression of heterogeneous population in breast cancer with significant correlation to their prognosis.

## Discussion

In this study, we proposed a novel method, TAPINTO, that can predict potential TAAs despite heterogeneous expression in cancer patient samples. Compared with other DEG-based methods, TAPINTO is the first method that can objectively identify outliers without using normal samples for comparison and can further identify TAAs from multiple transcriptomic profiles. For clinical analysis, the combined predicted TAAs could also serve as a signature for predicting the survival prognosis of heterogenous breast cancer, and the TAPINTO parameters were correlated with the therapeutic effects of antibody drugs in clinical trials. Moreover, TAPINTO provided information about tumor heterogeneity with distinct cancer pathways and disease types. Therefore, TAPINTO may become a useful tool in cancer research for academia and therapeutic studies.

As previously described, HGE occurs naturally in both tumor and normal samples from cancer patients. Thus, the reasonable selection of samples with overexpression is a crucial step for TAA identification. TAPINTO objectively identifies outliers that have higher expression than other tumor samples by detecting expression differences from the whole population. With a long-tailed distribution, a subgroup of samples is far from the central part of the distribution, hinting that there might be substantial differences in expression in 2 heterogeneous tumor subgroups (the high-expression subgroup and the normal-like subgroup) from that in the whole population. When an increasing number of low-expression samples were included and assessed, the SD (dispersion) was smaller than that previously calculated (red line, Fig. 3A), leading to the appearance of a turning point. Hence, we could use the maximum turning point to separate the 2 different subgroups, defined as the outlier subgroup and the central closed subgroup, with this method. Next, the ability of TAPINTO to identify overexpression was validated by IHC staining for HER2, and the cutoff value for outlier definition determined based on transcriptomic expression was mostly consistent with that determined based on protein expression. A highly accurate protein status was obtained based on the objective threshold, and the correlation between mRNA and protein fit with the expected values, which is usually observed in biological systems [41]. Thus, TAPINTO could identify samples with overexpression without assessment of adjacent normal samples (Fig. 3A and B). This advantage is due to the identification of a long-tailed distribution by TAPINTO and could avoid the bias of TAA prediction strategies that consider adjacent normal samples with a preneoplastic state [42]. In addition, in the identification of well-known TAAs, TAPINTO provided higher AUC values (over 0.86) than other methods, which achieved AUC values below 0.76 (Fig. 4).

TAPINTO provides 3 important parameters: gene expression level, frequency of overexpression, and FC to evaluate potential TAAs. With alluvial analysis, the TAA candidates can be assessed via TAA indexes. For example, ERBB2 and ESR1, as well-known TAAs of breast cancer, scored 6 and 3 points, respectively, for their potential as TAAs (Fig. 5A). Similar to ERBB2, CXCL9, KCNJ3, SQLE, MMP11, and SLC7A2 had the highest scores (6 points) and were regarded as potential TAAs (Table 2). It is worth noting that these 5 genes are closely correlated with tumor development (Table 3). CXCL9, as a chemokine, plays a role in metastasis in breast cancer [43]. KCNJ3 is overexpressed in ER^+^ breast cancer and regulates the metastasis of breast cancer [44,45]. SQLE is highly expressed in luminal A, ER^−^/HER2^+^ breast cancer and ER^+^ early-stage breast cancer and is associated with aggressive tumors such as TNBC [46–48]. MMP11 is overexpressed in invasive and metastatic breast cancer [49–51]. SLC7A2 is a cationic amino acid transporter, and its up-regulation may be associated with the development of breast cancer and resistant melanoma [52–54]. According to the published literature, these 5 candidates (CXCL9, KCNJ3, SQLE, MMP11, and SLC7A2) play a role in the tumorigenesis of distinct breast cancer subtypes, suggesting that TAPINTO can indeed identify potential TAAs based on the analysis of heterogeneous transcriptome expression. This advantage is particularly evident when compared with traditional methods: TAPINTO achieved AUC values exceeding 0.86 for both RTK and non-RTK antigen families, substantially outperforming LIMMA (AUC < 0.76) and DIDS (AUC < 0.66) (Fig. 4). These performance gains reflect the fundamental limitation of mean-based approaches such as LIMMA and *t* test, which are designed for homogeneous populations and therefore dilute the signal from the overexpressing subgroup when the majority of tumor samples exhibit normal-like expression levels. By contrast, TAPINTO directly targets this overexpressing subgroup through SD-based outlier detection, enabling more precise TAA identification in heterogeneous populations. Moreover, their integrated expression as a signature could predict the survival risk of the whole breast cancer population with heterogeneity (Fig. 6C), which further validated their oncogenic roles in the complex regulation of tumor progression. It can demonstrate that a tumor with heterogeneity is more likely to depend on a group of critical oncogenes than a single one to process the complexity of oncogenic signaling [55].

Furthermore, the alluvial analysis of TAPINTO indicated that TAA parameters might be associated with therapeutic effects in clinical trials. For example, the 3 TAPINTO parameters (AvExp, Freq, and FC) of well-known therapeutic targets in breast cancer samples in the clinical trials (VEGF and PD-L1) were not only significantly different from the respective values in adjacent normal samples (adjusted *P* < 0.05) but also related to clinical outcomes (PFS, ORR, and SAE) (Fig. 5B and Table 1). In a publication by Cheever et al. [14], a panel of content experts also suggested that there are many features of antigen candidates, including the expression level and frequency of antigen-positive patients, that are associated with therapeutic effects and could be used to rank ideal cancer antigens for tumor vaccine development. Figure 5B and Table 1 reveal that the frequency, AvExp, and FC of VEGF are all relatively moderate, and they were related with clinical outcome: 32.2% ORR, 9.2 months PFS, and 37.57% SAEs. However, CD274 had a low frequency, low AvExp, and high FC, which were also consistent with clinical outcomes: 9.6% ORR, 2.1 months PFS, and 20% SAEs. Therefore, TAPINTO can not only predict TAAs but also be used as a tool to evaluate the therapeutic effects of drugs targeting the predicted TAAs. Our study is the first method to directly implicate the relationship between these features and clinical outcomes from the perspective of transcriptomic data.

Moreover, the TAA score derived from TAPINTO can also be used to identify oncogenic pathways based on enriched gene sets. In the GSEA, TAPINTO interestingly identified enrichment of pathways related to sex hormones, which is a characteristic of the subgroup of invasive breast cancer patients who have overexpression of estrogen or androgen [56]. Dramatically, the oncogenic hallmarks of angiogenesis, apoptosis, EMT, hypoxia, DNA repair, and the G2/M checkpoint, which have been explored by published literature, were identified by the TAA score (Fig. 6A). Indeed, it has been proposed that overexpressed MAPs usually trigger many cancer hallmarks, such as proliferation, angiogenesis, and metastasis [1–4,57]. Therefore, the TAA score could be applied to identify not only TAAs but also potential oncogenic pathways. In addition to functional enrichment analysis, DOSE based on the TAA score also identified distinct subtypes of breast cancer, such as distinct pathologic types (sporadic breast carcinoma, ductal breast carcinoma, basal-like breast carcinoma, and contralateral breast cancer), stages (benign breast neoplasm, stage 0 breast carcinoma, early-stage breast cancer, and malignant neoplasm), and molecular type (triple-negative breast carcinoma and familial breast cancer)*.* These results demonstrate that TAPINTO can be more suitable than other methods for predicting TAAs for a specific heterogeneous subpopulation based on complex and heterogeneous expression profiles (Fig. 6B).

Despite its efficacy, TAPINTO has certain limitations. To minimize false positives arising from technical noise or biological variability, the algorithm requires candidates to satisfy multiple thresholds across Freq, AvExp, and FC parameters. This multi-dimensional filtering acts as a safeguard against transient expression spikes. However, as TAPINTO currently operates on transcriptomic data, the predicted TAAs require further validation at the proteomic level to account for posttranslational modifications. Future iterations incorporating proteomic or human leukocyte antigen (HLA)-peptidomic datasets could further enhance the precision of TAA identification. Finally, the current study addresses interpatient tumor heterogeneity using bulk RNA-seq data; applying TAPINTO to multisector or longitudinal RNA-seq datasets with matched clinical outcomes would be a valuable future direction to assess its utility in capturing intratumoral heterogeneity and predicting response to targeted therapy.

In conclusion, the heterogeneous features of malignant tumors result in aberrant gene expression affecting different molecular pathways, which leads to diverse susceptibilities to clinical therapy [58,59]. Therefore, TAPINTO can be used to advance personalized precision medicine for heterogeneous cancer patients by enabling exploration of potential TAAs and their related pathways. In addition, TAPINTO-identified potential targets may improve the efficiency of novel drug development, especially for drug combinations, which are being increasingly used to overcome intratumor heterogeneity within patients who bear a tumor bulk with a complex composition [60–62]. Critically, the demonstrated correlation between TAPINTO parameters and clinical outcomes—including ORR, PFS, and SAEs—suggests that TAPINTO could serve as a preclinical screening tool to prioritize TAA candidates before committing to costly and time-consuming clinical trials, thereby improving the probability of trial success. Furthermore, by stratifying patients based on antigen overexpression frequency, TAPINTO offers a transcriptomics-based framework for patient selection that may complement existing biomarker-driven enrollment strategies in precision oncology. Therefore, TAPINTO will be an ideal tool to improve personalized precision medicine for patients with highly heterogeneous tumors in the future.

## Data Availability

The RNA-seq data used in this study, corresponding to 1,109 BRCA samples and 113 adjacent normal samples, were obtained from The Cancer Genome Atlas (TCGA) database. All code used for the analysis is publicly available on GitHub at https://github.com/charleschuang1993/tapinto.git.
