# Supplementary material for: TAPINTO: A Novel Algorithm for Tumor-Associated Antigen Prediction Based on Information about Target Overexpression
Source: Comput Struct Biotechnol J. 2026 May 15;35(1):0109. doi: 10.34133/csbj.0109 (PMC13176607; doi:10.34133/csbj.0109)
Supplement: Supplementary 1 — Fig. S1 Tables S1 and S2 [file csbj.0109.f1.docx]

**Supplementary Table 1. Well-known TAAs in breast cancer.**

To verify the outlier detection ability of TAPINTO, 31 well-known TAAs of breast cancer were reviewed (as shown below). The list was constructed by literature research.

| **Ensembl ID** | **Gene** | **Protein** | **Description** | **Ref.** |
| --- | --- | --- | --- | --- |
| ENSG00000146648 | *EGFR* | EGFR | Epidermal growth factor receptor | [1, 2] |
| ENSG00000141736 | *ERBB2* | HER2 | erb-b2 receptor tyrosine kinase 2 | [3] |
| ENSG00000077782 | *FGFR1* | FGFR1 | Fibroblast growth factor receptor 1 | [4] |
| ENSG00000066468 | *FGFR2* | FGFR2 | Fibroblast growth factor receptor 2 | [4] |
| ENSG00000068078 | *FGFR3* | FGFR3 | Fibroblast growth factor receptor 3 | [4] |
| ENSG00000160867 | *FGFR4* | FGFR4 | Fibroblast growth factor receptor 4 | [4] |
| ENSG00000140443 | *IGF1R* | IGF1R | Insulin-like growth factor 1 receptor | [5] |
| ENSG00000105976 | *MET* | HGFR | Hepatocyte growth factor receptor | [6] |
| ENSG00000102755 | *FLT1* | VEGFR1 | Vascular endothelial growth factor receptor 1 | [7] |
| ENSG00000128052 | *KDR* | VEGFR2 | Vascular endothelial growth factor receptor 2 | [8] |
| ENSG00000037280 | *FLT4* | VEGFR3 | Vascular endothelial growth factor receptor 3 | [9, 10] |
| ENSG00000105388 | *CEACAM5* | CEA | Carcinoembryonic antigen-related cell adhesion molecule 5 | [11] |
| ENSG00000086548 | *CEACAM6* | CEACAM6 | Carcinoembryonic antigen-related cell adhesion molecule 6 | [12] |
| ENSG00000157240 | *FZD1* | Frizzled-1 | Frizzled class receptor 1 | [13] |
| ENSG00000111432 | *FZD10* | Frizzled-10 | Frizzled class receptor 10 | [13] |
| ENSG00000164930 | *FZD6* | Frizzled-6 | Frizzled class receptor 6 | [13] |
| ENSG00000155760 | *FZD7* | Frizzled-7 | Frizzled class receptor 7 | [13] |
| ENSG00000177283 | *FZD8* | Frizzled-8 | Frizzled class receptor 8 | [13] |
| ENSG00000196611 | *MMP1* | Interstitial collagenase | Matrix metalloproteinase-1 | [14] |
| ENSG00000099953 | *MMP11* | Stromelysin-3 | Matrix metalloproteinase-11 | [15] |
| ENSG00000157227 | *MMP14* | MMP14 | Matrix metalloproteinase-14 | [16] |
| ENSG00000102996 | *MMP15* | MMP15 | Matrix metalloproteinase-15 | [17] |
| ENSG00000125966 | *MMP24* | MMP24 | Matrix metalloproteinase-24 | [18, 19] |
| ENSG00000008516 | *MMP25* | MMP25 | Matrix metallopeptidase-25 | [18, 20] |
| ENSG00000137673 | *MMP7* | Matrilysin | Matrix metallopeptidase-7 | [21] |
| ENSG00000100985 | *MMP9* | MMP9 | Matrix metallopeptidase-9 | [22] |
| ENSG00000185499 | *MUC1* | Mucin-1 | Mucin 1, cell surface associated | [23] |
| ENSG00000173702 | *MUC13* | Mucin-13 | Mucin 13, cell surface associated | [24, 25] |
| ENSG00000169550 | *MUC15* | Mucin-15 | Mucin 15, cell surface associated | [26] |
| ENSG00000181143 | *MUC16* | Mucin-16 | Mucin 16, cell surface associated | [27] |
| ENSG00000145113 | *MUC4* | Mucin-4 | Mucin 4, cell surface associated | [28] |

**Supplementary Table 2. Therapeutic targets and treatment outcomes of clinical trials in breast cancer.**

The table shows clinical trial information to evaluate the association between TAPINTO parameters and clinical outcomes. The listed TAAs are common targets for breast cancer antibody treatment that have been tested in phase 3 or 4 clinical trials utilizing the same outcomes (ORR, PFS, and SAEs).

| **NCT number** | **Protein** | **Gene** | **Drug name** | **ORR (%)** | **PFS (month)** | **SAEs (%)** | **Phase** |
| --- | --- | --- | --- | --- | --- | --- | --- |
| NCT02555657 | PDL1 | CD274 | Pembrolizumab (anti-PD1 antibody) | 9.6 | 2.1 | 20% | 3 |
| NCT00373256 | VEGFA | VEGF | Bevacizumab | 32.2 | 9.2 | 37.57% | 3 |
| NCT00703326 | VEGFR2 | KDR | Ramucirumab | 44.7 | 9.5 | 37.23% | 3 |
| NCT01301729 | HER2 | ERBB2 | Trastuzumab | 81.3 | 9.9 | 15.63% | 4 |
| ORR: objective response rate (%); PFS: progression-free survival (month); SAEs: serious adverse events (%) | | | | | | | |

*** [Available, active, not recruiting, completed, suspended, terminated, or withdrawn] | Studies With Results | Interventional Studies | Breast Cancer | Phase 3, 4**

| **Supplementary Figure 1. A. Oncogene-related pathways:** Five well-known oncogene-regulated signaling pathways were identified as enriched in GSEA according to TAPINTO parameters: *IL2_STAT5_SIGNALING*, *KRAS_SIGNALING_UP*, *MTORC1_SIGNALING*, *MYC_TARGET_V1*, and *P53_PATHWAY*. **B.** **Hormone-related response:** Three well-known hormone response-related gene sets were identified as enriched in GSEA according to TAPINTO parameters: *ANDROGEN_RESPONSE*, *ESTROGEN_RESPONSE_EARLY*, and *ESTROGEN_RESPONSE_LATE*. **C. Environment-related pathway:** Three well-known environment-related gene sets were identified as enriched in GSEA according to TAPINTO parameters: *APICAL_SURFACE*, *COAGULATION*, and *PROTEIN_SECRETION*. **D. Metabolism-related pathway:** Four well-known metabolism-related gene sets were identified as enriched in GSEA according to TAPINTO parameters: *CHOLESTEROL_HOMEOSTASIS*, *FATTY_ACID_METABOLISM*, *GLYCOLYSIS,* and *XENOBIOTIC_METABOLISM*. **E.** **Damage response pathway:** Two well-known damage response-related gene sets were identified as enriched in GSEA according to TAPINTO parameters: *UNFOLDED_PROTEIN_RESPONSE* and *UV_RESPONSE_UP*. **F. Immune-related pathway:** Five well-known immune-related gene sets were identified as enriched in GSEA according to TAPINTO parameters: *ALLOGRAFT_REJECTION*, *COMPLEMENT*, *INTERFERON_ALPHA_RESPONSE*, *INTERFERON_GAMMA_RESPONSE*, and *TNFA_SIGNALING_VIA_NFKB*.  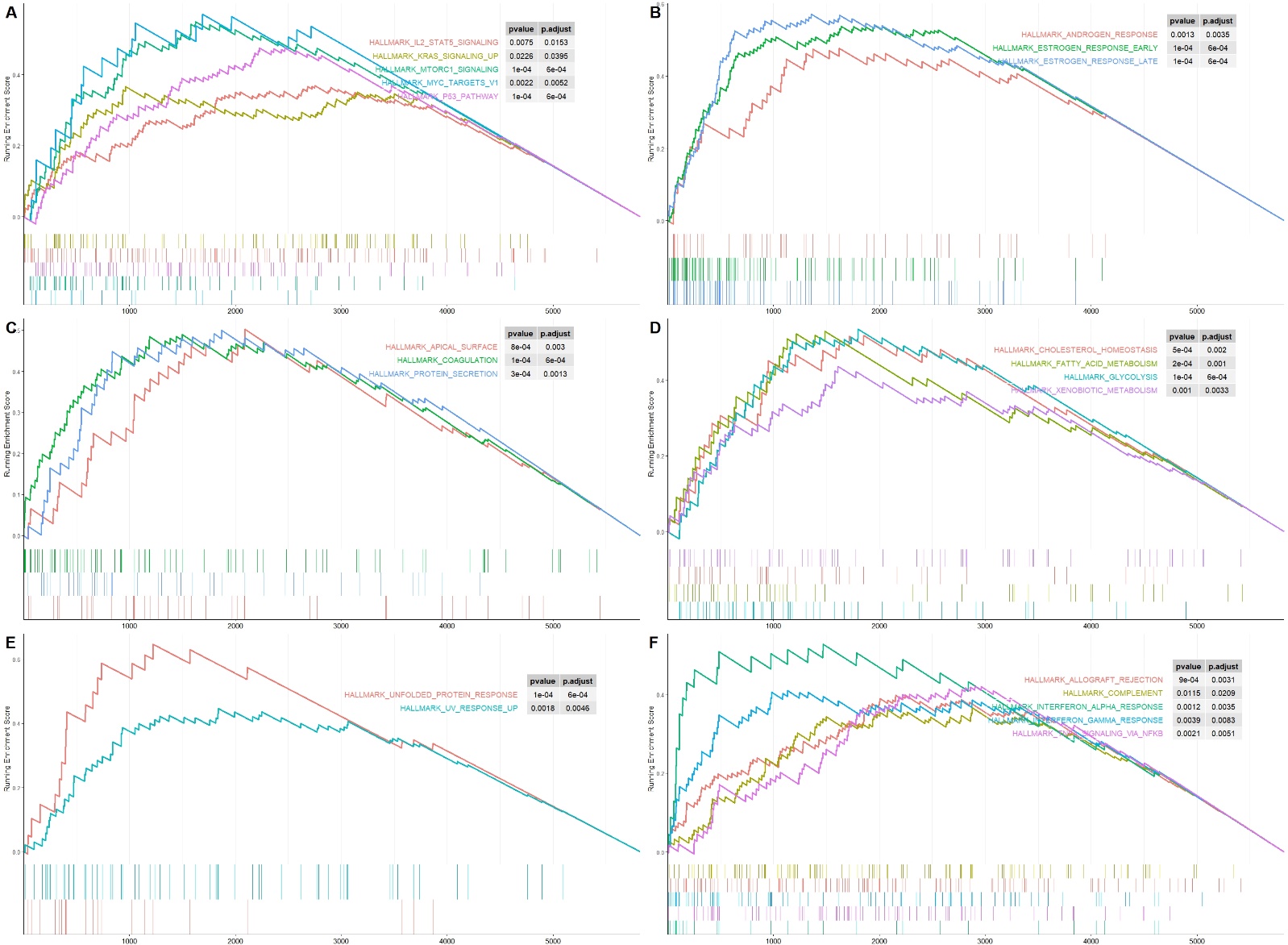 |
| --- |

**Supplementary Reference**

1. Rimawi, M.F., et al., *Epidermal growth factor receptor expression in breast cancer association with biologic phenotype and clinical outcomes.* Cancer, 2010. **116**(5): p. 1234-42.

2. Maennling, A.E., et al., *Molecular Targeting Therapy against EGFR Family in Breast Cancer: Progress and Future Potentials.* Cancers (Basel), 2019. **11**(12).

3. Oh, D.Y. and Y.J. Bang, *HER2-targeted therapies - a role beyond breast cancer.* Nat Rev Clin Oncol, 2020. **17**(1): p. 33-48.

4. Perez-Garcia, J., et al., *Targeting FGFR pathway in breast cancer.* Breast, 2018. **37**: p. 126-133.

5. Ekyalongo, R.C. and D. Yee, *Revisiting the IGF-1R as a breast cancer target.* NPJ Precis Oncol, 2017. **1**.

6. Ho-Yen, C.M., J.L. Jones, and S. Kermorgant, *The clinical and functional significance of c-Met in breast cancer: a review.* Breast Cancer Res, 2015. **17**: p. 52.

7. Ceci, C., et al., *Role of VEGFs/VEGFR-1 Signaling and its Inhibition in Modulating Tumor Invasion: Experimental Evidence in Different Metastatic Cancer Models.* Int J Mol Sci, 2020. **21**(4).

8. Zhang, S., et al., *Immunoglobulin-like domain 4-mediated ligand-independent dimerization triggers VEGFR-2 activation in HUVECs and VEGFR2-positive breast cancer cells.* Breast Cancer Res Treat, 2017. **163**(3): p. 423-434.

9. Varney, M.L. and R.K. Singh, *VEGF-C-VEGFR3/Flt4 axis regulates mammary tumor growth and metastasis in an autocrine manner.* Am J Cancer Res, 2015. **5**(2): p. 616-28.

10. Su, J.L., et al., *The role of the VEGF-C/VEGFR-3 axis in cancer progression.* Br J Cancer, 2007. **96**(4): p. 541-5.

11. Powell, E., et al., *A functional genomic screen in vivo identifies CEACAM5 as a clinically relevant driver of breast cancer metastasis.* NPJ Breast Cancer, 2018. **4**: p. 9.

12. Lewis-Wambi, J.S., et al., *Overexpression of CEACAM6 promotes migration and invasion of estrogen-deprived breast cancer cells.* Eur J Cancer, 2008. **44**(12): p. 1770-9.

13. Zeng, C.M., Z. Chen, and L. Fu, *Frizzled Receptors as Potential Therapeutic Targets in Human Cancers.* Int J Mol Sci, 2018. **19**(5).

14. Wang, Q.M., et al., *MMP-1 is overexpressed in triple-negative breast cancer tissues and the knockdown of MMP-1 expression inhibits tumor cell malignant behaviors in vitro.* Oncol Lett, 2019. **17**(2): p. 1732-1740.

15. Gonzalez de Vega, R., et al., *MMP-11 as a biomarker for metastatic breast cancer by immunohistochemical-assisted imaging mass spectrometry.* Anal Bioanal Chem, 2019. **411**(3): p. 639-646.

16. Hillebrand, L.E., et al., *MMP14 empowers tumor-initiating breast cancer cells under hypoxic nutrient-depleted conditions.* FASEB J, 2019. **33**(3): p. 4124-4140.

17. Radisky, E.S. and D.C. Radisky, *Matrix metalloproteinases as breast cancer drivers and therapeutic targets.* Front Biosci (Landmark Ed), 2015. **20**: p. 1144-63.

18. Benson, C.S., et al., *Expression of matrix metalloproteinases in human breast cancer tissues.* Dis Markers, 2013. **34**(6): p. 395-405.

19. Kohrmann, A., et al., *Expression of matrix metalloproteinases (MMPs) in primary human breast cancer and breast cancer cell lines: New findings and review of the literature.* BMC Cancer, 2009. **9**: p. 188.

20. Hegedus, L., et al., *Additional MDA-MB-231 breast cancer cell matrix metalloproteinases promote invasiveness.* J Cell Physiol, 2008. **216**(2): p. 480-5.

21. Sizemore, S.T., et al., *Hypomethylation of the MMP7 promoter and increased expression of MMP7 distinguishes the basal-like breast cancer subtype from other triple-negative tumors.* Breast Cancer Res Treat, 2014. **146**(1): p. 25-40.

22. Moirangthem, A., et al., *Simultaneous knockdown of uPA and MMP9 can reduce breast cancer progression by increasing cell-cell adhesion and modulating EMT genes.* Sci Rep, 2016. **6**: p. 21903.

23. Maeda, T., et al., *MUC1-C Induces PD-L1 and Immune Evasion in Triple-Negative Breast Cancer.* Cancer Res, 2018. **78**(1): p. 205-215.

24. Filippou, P.S., et al., *Exploring the potential of mucin 13 (MUC13) as a biomarker for carcinomas and other diseases.* Clin Chem Lab Med, 2018. **56**(11): p. 1945-1953.

25. Sheng, Y.H., et al., *MUC13 protects colorectal cancer cells from death by activating the NF-kappaB pathway and is a potential therapeutic target.* Oncogene, 2017. **36**(5): p. 700-713.

26. Jung, K., et al., *Profiling gene promoter occupancy of Sox2 in two phenotypically distinct breast cancer cell subsets using chromatin immunoprecipitation and genome-wide promoter microarrays.* Breast Cancer Res, 2014. **16**(6): p. 470.

27. Lakshmanan, I., et al., *MUC16 induced rapid G2/M transition via interactions with JAK2 for increased proliferation and anti-apoptosis in breast cancer cells.* Oncogene, 2012. **31**(7): p. 805-17.

28. Mercogliano, M.F., et al., *Invasive micropapillary carcinoma of the breast overexpresses MUC4 and is associated with poor outcome to adjuvant trastuzumab in HER2-positive breast cancer.* BMC Cancer, 2017. **17**(1): p. 895.
